# Supplementary material for: Integrated analysis of the aqueous humor microbiome and lens capsule transcriptome in high myopia cataract: a pilot study
Source: Front Med (Lausanne). 2026 Jun 16;13:1845205. doi: 10.3389/fmed.2026.1845205 (PMC13314463; doi:10.3389/fmed.2026.1845205)
Supplement: Supplementary file 3 [file Data_Sheet_1.zip › 7.Average/2.Alpha_Diversity/rank_abundance/C372089/RankAbundance.pdf]

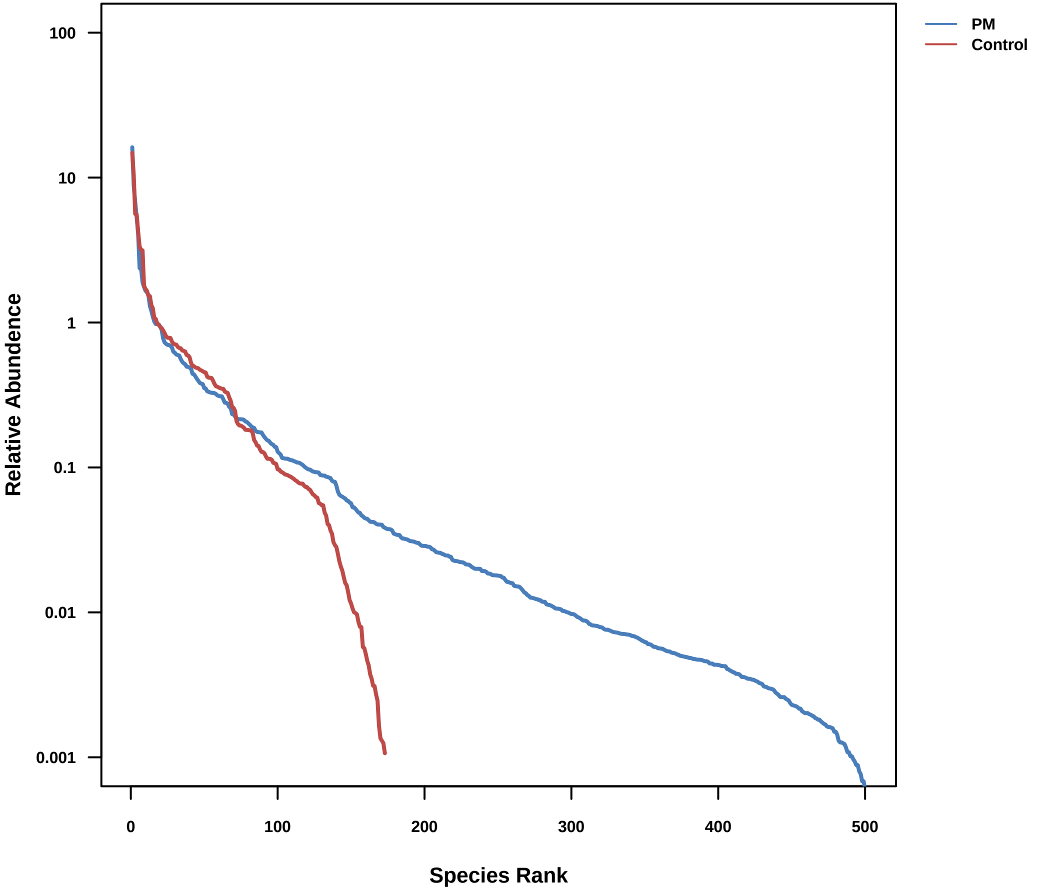

PM
Control
Relative Abundence
100
10
1
0.1
0.01
0.001
0
100
200
300
400
500
Species Rank
